# Mail merge can be used to create personalized questionnaires in complex surveys

**DOI:** 10.1186/s13104-015-1570-5

**Published:** 2015-10-16

**Authors:** Monica Taljaard, Shazia Hira Chaudhry, Jamie C. Brehaut, Charles Weijer, Jeremy M. Grimshaw

**Affiliations:** Clinical Epidemiology Program, Ottawa Hospital Research Institute, Ottawa Hospital, 1053 Carling Avenue, Civic Campus, Admin Services Building, C409, ASB 2-004, Civic Box 693, Ottawa, ON K1Y 4E9 Canada; Department of Epidemiology and Community Medicine, University of Ottawa, Ottawa, ON Canada; Rotman Institute of Philosophy, Western University, London, ON Canada; Department of Medicine, University of Ottawa, Ottawa, ON Canada; Department of Medicine, Western University, London, ON Canada; Department of Epidemiology and Biostatistics, Western University, London, ON Canada; Clinical Epidemiology Program, Ottawa Hospital Research Institute, The Ottawa Hospital, General Campus, 501 Smyth Road, Box 711, Ottawa, ON K1H 8L6 Canada

**Keywords:** Questionnaire personalization, Question comprehension, Web survey, Response rate, Survey methodology, Measurement error

## Abstract

**Background:**

Low response rates and inadequate question comprehension threaten the validity of survey results. We describe a simple procedure to implement personalized—as opposed to generically worded—questionnaires in the context of a complex web-based survey of corresponding authors of a random sample of 300 published cluster randomized trials. The purpose of the survey was to gather more detailed information about informed consent procedures used in the trial, over and above basic information provided in the trial report. We describe our approach—which allowed extensive personalization without the need for specialized computer technology—and discuss its potential application in similar settings.

**Results:**

The mail merge feature of standard word processing software was used to generate unique, personalized questionnaires for each author by incorporating specific information from the article, including naming the randomization unit (e.g., family practice, school, worksite), and identifying specific individuals who may have been considered research participants at the cluster level (family doctors, teachers, employers) and individual level (patients, students, employees) in questions regarding informed consent procedures in the trial. The response rate was relatively high (64 %, 182/285) and did not vary significantly by author, publication, or study characteristics. The refusal rate was low (7 %).

**Conclusion:**

While controlled studies are required to examine the specific effects of our approach on comprehension, quality of responses, and response rates, we showed how mail merge can be used as a simple but useful tool to add personalized fields to complex survey questionnaires, or to request additional information required from study authors. One potential application is in eliciting specific information about published articles from study authors when conducting systematic reviews and meta-analyses.

## Background

The design of clear and comprehensible questionnaires is essential in promoting survey validity, but this can be challenging when the subject matter is complex and the target population heterogeneous. Ambiguous wording may decrease response and item completion rates due to perceived difficulty of questions and respondent frustration or fatigue; it can also increase measurement error due to poor question comprehension. As part of a larger study examining ethical issues in cluster randomized trials [1, 2], we reviewed a random sample of 300 cluster randomized trials in health research published between 2000 and 2008 [3]. Due to the paucity of specific information about ethical issues reported in the sample of trials [4], we designed a survey to gather more detailed information from the corresponding authors. The survey was complex: it aimed to gather specific information about the presence of any cluster “gatekeepers” or other individuals who were approached for permission to randomize clusters, as well as informed consent procedures administered to research participants at the cluster and/or individual level. Further details and results of the survey may be found elsewhere [5, 6]. We were faced with several challenges in designing our questionnaire, including diversity of the sample with respect to country and research area of study authors, potential lack of familiarity with the concept of cluster randomization, lack of standard definitions of “gatekeepers” and “research participants”, and diversity of key elements of the trials themselves including types of clusters (e.g., medical practices, schools, communities, work sites, sports teams), types of participants at the individual level and/or cluster level, and specific study interventions and data collection procedures that may or may not have required consent. These challenges made a traditional survey that presented questions in a uniform way problematic to operationalize.

To address these challenges, we considered using an interviewer-administered telephone survey, which would allow real-time clarifications to respondents. However, this was considered logistically infeasible given the sample size. A self-administered web-based questionnaire with the addition of a glossary of definitions was a second option, but would have considerably increased the length of the questionnaire without necessarily improving question comprehension. A third and preferred option was to generate a unique, personalized questionnaire for each sample member. This would allow us to customize the questions to each author by incorporating study-specific information from the published article, including naming the randomization unit or “cluster” (e.g., family practice, school, worksite, sports team), and referring to specific individuals who might be considered potential participants at the cluster level (e.g., health care providers, teachers, employers, coaches) and individual level (e.g., patients, students, employees, players). We expected that this would not only improve question comprehension leading to higher quality results, but also increase participation as the incorporation of personal information might increase saliency [7]. While telephone, face-to-face and web-based questionnaire customization has been used for decades at survey organizations, supported by advances in computer-assisted interviewing [8, 9], we were restricted to the use of a simple and cheap self-administered method that did not require specialized computer technology.

## Methods

To automate the process of creating the questionnaires, we explored use of the mail merge feature in a standard word processing package. Mail merge is a word processor function that creates personalized documents by inputting values for variables from a source database into a template. It is most commonly used to insert names and addresses, but in our study, we extended its use to questionnaire items. We first designed a questionnaire template, inserting “fields” in place of terms that we thought would be easier to comprehend if replaced by the study author’s own terminology rather than a generic term. We created a source database in the form of a spreadsheet, where each column corresponded to a field in the template. We reviewed the published articles, extracted the study-specific terms from the publication, and entered them into the spreadsheet. Unique questionnaires were then generated from merging the source database into the document template. A similar process was used to create personalized correspondence (i.e., survey invitation and reminder letters) identifying the title of the publication, journal title, and year of publication. In total, 19 fields were used to personalize the questionnaire. Table 1 presents examples of the generic and personalized terms that were used, as well as the total number of unique terms that were generated for our sample.

**Table 1:** Examples of items personalized in our survey questionnaire showing extent of personalization required to generate n = 285 unique questionnaires

Type of information	Generic wording	Personalized wording (based on target study publication)	Total number of unique fields
Study title, journal, and year of publication	Your study was randomly selected for this survey	Your study (A Cluster-Randomized Controlled Trial Evaluating the Effect of a Handwashing-Promotion Program in Chinese Primary Schools,) published in [Am J Trop Med Hyg (2007)] was randomly selected for this survey	285
Research ethics approval	Did you seek ethics approval to conduct the study?	[Our review of your paper indicates that research ethics approval was (sought) (not sought) to conduct the study. Is this correct?]	2
Type of committee	How many ethics committees did you approach?	How many [institutional review boards (IRBs)] [Research Ethics Boards (REBs)] [Research Ethics Committees (RECs)…] did you approach?	13
Study permission	Did one or more gatekeepers provide agreement or consent to the clusters’ involvement in your study?	Did any (school principals or school district administrators) provide agreement or consent to the involvement of (schools) in your study?	93
Any other permission	Did you seek approval from anyone else to conduct the study?	Did you seek approval from anyone else, such as: (Ministry of Education, Department of Public Health, or school advisory board,) to conduct the study?	16
Unit of randomization	Was consent sought before randomization of clusters?	Was consent sought before randomization of (schools)?	138
Cluster level participants	Did cluster-level participants in the intervention arm provide consent?	Did (the teachers) in your intervention arm consent to receiving the study interventions?	98
Individual level participants	Did individual level participants provide consent?	Did (parents or guardians of students) consent to the collection of data in your study?	113

We used cognitive interviewing [7, 10] to identify troublesome terms in the initial generic version, and to pre-test the personalized version. Participants for cognitive interviewing were primary authors of published cluster randomized trials, selected to represent a range of countries, types of interventions, and study settings. Fifteen individuals were invited to participate in a 45–60 min session and 11 agreed. Immediately prior to the session, we emailed the survey cover letter and a Word version of the questionnaire formatted as a fillable form. Participants were asked not to view the documents until the start of the session. As they completed the questionnaire, participants were asked to verbalize their thoughts and actions, from reading the questions aloud, to providing their final answers. Participants were also asked to share their overall impression of the questionnaire and survey. Following each interview, the survey questionnaire was modified iteratively to reflect any new understanding that had arisen. Comments from earlier think-aloud sessions that the questionnaire is, for example, “complicated to work through and read” contrasted with comments from later think-aloud sessions that the questionnaire is, for example, “nicely laid-out, easy to go through.”

The final questionnaire was operationalized into a secure web survey using MS Visual Studio 2005 and MS SQL Server 2000. Upon each successful login attempt, respondents were time-stamped in the survey database. Open text boxes were placed throughout so that respondents could clarify a response, or provide a written explanation. The web-based interface was tested on multiple platforms prior to full-scale implementation. The estimated final questionnaire completion time was 15–20 min. A pre-notification email was sent followed shortly by the survey invitation containing the survey URL, the title of the published study, unique password, and details of the survey incentive (a book on the design and analysis of cluster randomized trials). One week later, a thank you and reminder email was sent. Non-respondents were emailed a reminder 2 weeks after the initial survey invitation, and thereafter were mailed a reminder letter by post. The response rate was calculated according to the American Association for Public Opinion Research (AAPOR) guideline for web surveys of specifically named persons [11]. We tabulated response rates by author, publication, and study characteristics thought to be associated with an increased risk of nonresponse, and tested the statistical significance of differences using Chi squared or Wilcoxon two-sample tests.

## Results and discussion

**Fig. 1:**
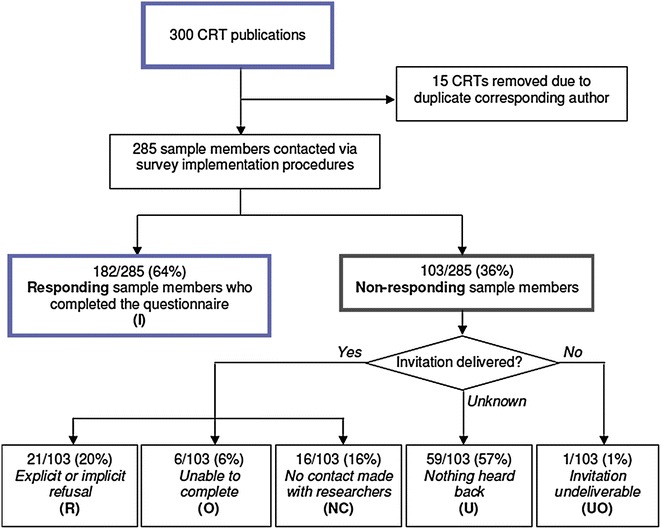
Survey response rate calculation using American Association for Public Opinion Research guidelines. The survey response rate was 64 %. This minimal response rate calculation includes sample members who completed the survey (I), over all invited sample members, including all category (U) sample members for whom it is unknown whether the survey invitation reached them. These sample members are included in the denominator of the response rate because their contact details were confirmed and updated prior to survey implementation. It is estimated therefore that the majority of category (U) sample members received the survey invitation but chose not to respond

The characteristics of the respondents and non-respondents have been presented elsewhere [5]. Of the random sample of 300 trials, 15 were excluded to avoid having multiple publications by the same corresponding author in the sample. The trials were diverse, covering a wide range of countries and settings in health research. The calculation of the response rate according to AAPOR guidelines is presented in Fig. 1. The overall response rate was 64 % (182/285). There were no important differences in response rates among the subgroups examined: response rates were similar among primary authors from economically developed and developing countries (p = 0.39), by year of publication (p = 0.36), by journal impact factor (p = 0.95) and among studies conducted in health care organization settings (primary and hospital) compared to studies conducted in public health and health promotion (e.g., schools, community-settings) (p = 0.43). The refusal rate for the survey, both explicit and implicit, was 7 %. (Implicit refusals consisted of sample members who did not submit the questionnaire but were time stamped through the web server as having logged into the survey at least once.) Among the open text box comments received from sample members, only two expressed difficulty related to understanding the questionnaire.

## Conclusions

Response rates to surveys are generally declining, particularly among healthcare practitioners. While questionnaire personalization has been used for many years in survey research, and experimental effects of computerization and instrument customization on survey outcomes has been studied using more rigorous research designs (e.g., [12–14]), web-based questionnaires are still scarce in Epidemiological research [15], and we are unaware of previous studies that have used simple mail merge to customize a complex survey questionnaire within the setting of a systematic review. We examined the potential impact of our approach by considering feedback from participants, the survey response rate, implicit and explicit refusal, and variability of response rates across publication, study, and author characteristics. We expected that questionnaire personalization might facilitate questionnaire comprehension, reduce potential points of frustration, and reduce barriers to response and completion: although we were unable to rigorously evaluate its impact within the scope of our study, we received no negative feedback and only two requests for clarification from participants despite the high degree of complexity of the survey topic and diversity of the sample. Although the survey was relatively long, the response rate was 64 %—higher than the average response rate of 58 % found in a review of surveys of health professionals [16]. Given that this was an international survey of busy researchers, whose studies may have been published several years earlier and whose first language may not have been English, this response rate was considered acceptable. Response rates were similar across countries of the primary author as well as various publication and study characteristics.

While we believe that personalization was a factor in our successful response rate, we were unable to use experimental manipulation to examine its effects more rigorously. In particular, we lacked a control group that presented respondents with the alternative generic question language and therefore cannot attribute our success solely to this aspect of our survey. It is possible that other features of the survey, including the personalized invitations and the composition of our study population (researchers and scientists) facilitated questionnaire completion. Our results, therefore, are merely descriptive; future work should involve stronger designs to determine the effect of questionnaire personalization on survey response and measurement error, and in different populations. Nevertheless, Table 1 provides an indication of the extent of personalization required in our survey: without the provision of these study-specific terms, respondents would have been required to make the conversions from the generic term to the study-specific term with substantially increased risk of error and misunderstanding.

We believe that our simple approach to questionnaire personalization may be useful in other settings where resource constraints limit access to more sophisticated technology, where surveyors have access to prior information about sample members or where a sample is diverse and terminology differs across settings. For example, when conducting systematic reviews and meta-analyses of original research studies [17], inadequate reporting of study procedures and outcomes in publications often necessitates contacting study authors to obtain missing information. Contacting study authors is encouraged because syntheses of incomplete data can lead to biased estimates [18, 19]. A common method is email solicitation, where data collection forms are sent by email attachment. To promote higher response rates and improve response accuracy, researchers may consider the use of the mail merge feature to add personalized fields to data collection forms. Future research studies could consider the use of experimentation to rigorously examine the effects of this approach and to explore improved comprehension versus saliency as plausible pathways to improved response.

## References

[1] Taljaard M, Weijer C, Grimshaw JM, BelleBrown J, Binik A, Boruch R, Brehaut JC, Chaudhry SH, Eccles MP, McRae A, Saginur R, Zwarenstein M, Donner A (2009). Ethical and policy issues in cluster randomized trials: rationale and design of a mixed methods research study. Trials.

[2] Weijer C, Grimshaw JM, Taljaard M, Binik A, Boruch R, Brehaut JC, Donner A, Eccles MP, Gallo A, McRae AD, Saginur R, Zwarenstein M (2011). Ethical issues posed by cluster randomized trials in health research. Trials.

[3] Taljaard M, McGowan J, Grimshaw JM, Brehaut JC, McRae A, Eccles MP, Donner A (2010). Electronic search strategies to identify reports of cluster randomized trials in MEDLINE: Low precision will improve with adherence to reporting standards. BMC Med Res Methodol.

[4] Taljaard M, McRae AD, Weijer C, Bennett C, Dixon S, Taleban J, Skea Z, Brehaut J, Eccles MP, Donner A, Saginur R, Boruch RF, Grimshaw JM (2011). Inadequate reporting of research ethics review and informed consent in cluster randomized trials: review of a representative sample of published trials. BMJ.

[5] Chaudhry SH, Brehaut JC, Grimshaw JM, Weijer C, Boruch R, Donner A, Eccles MP, McRae AD, Saginur R, Skea ZA, Zwarenstein M, Taljaard M (2013). Challenges in the research ethics review of cluster randomized trials: International survey of investigators. Clin Trials.

[6] Taljaard M, Chaudhry SH, Brehaut JC, Weijer C, Boruch R, Donner A, Eccles MP, McRae AD, Saginur R, Zwarenstein M, Grimshaw JM (2014). Survey of consent practices in cluster randomized trials: improvements are needed in ethical conduct and reporting. Clin Trials.

[7] Dillman DA, Smyth JD, Christian LM (2009). Internet, mail, and mixed-mode surveys : the tailored design method.

[8] Couper MP, Baker RP, Bethlehem J, Clark CZF, Martin J, Nicholls II WL, O’Reilly J (editors) Computer Assisted Survey Information Collection. New York: Wiley; 1998.

[9] Couper MP (2008). Designing Effective Web Surveys.

[10] Collins D (2003). Pretesting survey instruments: an overview of cognitive methods. Qual Life Res.

[11] The American Association for Public Opinion Research. Standard Definitions: Final dispositions of case codes and outcome rates for surveys. 7th ed. 2011.

[12] Lynn P, Engel U, Jann B, Lynn P, Scherpenzeel A, Sturgis P (2014). Targeted response inducement strategies on longitudinal surveys. Improving Survey Methods: Lessons from Recent Research.

[13] Jäckle A. Dependent Interviewing: A Framework and Application to Current Research. In: Lynn P, editor. Methodology of Longitudinal Surveys. Chichester: Wiley. 2009. doi:10.1002/9780470743874.ch6.

[14] Schouten B, Calinescu M, Luiten A (2013). Optimizing quality of response through adaptive survey designs. Surv Methodol.

[15] van Gelder MM, Bretveld RW, Roeleveld N (2010). Web-based questionnaires: the future in epidemiology?. Am J Epidemiol.

[16] Galea S, Tracy M (2007). Participation rates in epidemiologic studies. Ann Epidemiol.

[17] Mullan RJ, Flynn DN, Carlberg B, Tleyjeh IM, Kamath CC, LaBella ML, Erwin PJ, Guyatt GH, Montori VM (2009). Systematic reviewers commonly contact study authors but do so with limited rigor. J Clin Epidemiol.

[18] Chan AW, Altman DG (2005). Identifying outcome reporting bias in randomised trials on PubMed: review of publications and survey of authors. BMJ.

[19] Higgins JPT, Green S (editors). Cochrane handbook for systematic reviews of interventions Version 5.0.2 [updated September 2009]. The Cochrane Collaboration. Retrieved from www.cochrane-handbook.org. 2009.

